# Application of neuroendoscopy in the surgical treatment of complicated hemifacial spasm

**DOI:** 10.17712/nsj.2017.1.20150567

**Published:** 2017-01

**Authors:** Ming Zhi, Xiao J. Lu, Qing Wang, Bing Li

**Affiliations:** *From the Department of Neurosurgery, Hospital Affiliated with Nanjing Medical University of Wuxi, Wuxi, China*

## Abstract

**Objective:**

To explore the value of neuroendoscopy in surgery for primary hemifacial spasm (HFS) in patients with complicated local anatomy.

**Methods:**

Endoscopic-assisted microvascular decompression (MVD) was performed in 42 patients with HFS with complicated local anatomy from January 2008 to January 2012 in our department, in the event of a significant blind spot, endoscopic exploration was performed with multi-angle 360-degree observation, including exploration of the brainstem facial nerve root exit zone (REZ) and exploration of the distal end of the nerve, and the relationships between blood vessels and nerves were carefully determined. After surgery, endoscopic examination was performed again to rule out vascular omissions, avascular excessive stretch, kinking, or formation of new compressions. The relevant data of all cases were retrospectively analyzed.

**Results:**

All patients were followed for 18-30 months, 41 patients had complete remission without recurrence (97.6%), 3 cases recovered to grade 0 from discharge grade I, 1 case of hearing loss was fully restored in 6 months, and 1 case of grade II was not significant increased to the end of follow-up.

**Conclusions:**

Neuroendoscopy is an effective supplement to traditional MVD in treating HFS. In particular, in patients with complicated or abnormal local anatomy (for example small posterior fossa volume, abnormal fullness of the cerebellar flocculus, petrous bone block, local thickening of arachnoid adhesions, and unidentified offending vessels), neuroendoscopy can greatly improve the effectiveness of surgery.

Hemifacial spasm (HFS) is a common neurological condition. Traditional microvascular decompression (MVD) has been the main treatment for patients with HFS.^1^ However, in some HFS patients with more complex anatomy (for example small posterior fossa volume, abnormal fullness of the cerebellar flocculus, petrous bone block, local thickening of arachnoid adhesions, and unidentiied ofending vessels), simple MVD effect is not good^2^ because their congenitally limitations of viewing angle and lighting. Therefore, minimally invasive endoscopic surgical techniques have been increasingly used in recent years to treat HFS. In contrast to the microscope, the neuroendoscope provides excellent lighting and a panoramic viewing angle, and gradually become an effective complement to MVD for HFS.^3^,^4^ In our research we retrospectively analyzed the indication, the surgical technique, the advantages, and the disadvantages of the neuroendoscopy in 42 patients with HFS with complicated local anatomy during the period from January 2008 to January 2012 in the Department of Neurosurgery, Hospital Affiliated with Nanjing Medical University of Wuxi, Wuxi, China.

## Methods

### Clinical data

Forty-two HFS patients (18 males, 24 females; mean age, 48.2 years [range, 24–68 years]) were enrolled in this study during the period from Janurary 2008 to Janurary 2012 in the Department of Neurosurgery, Hospital Affiliated with Nanjing Medical University of Wuxi, Wuxi, China. The study was endorsed by the ethics committee of Nanjing Medical University, Wuxi, China. The disease course ranged from 0.8 to 5.2 years. The lesion was on the right in 26 cases and on the left in 16 cases. According to the Shorr grading system, there were 12 cases of grade II, 29 cases of grade III, and one case of grade IV HFS. Preoperative findings included abnormal fullness of the cerebellar flocculus (n=28), narrow posterior fossa (n=9), offending vessels in the outer peripheral portion of the facial nerve with protrusion of abnormal bone (n=3), complex vascular anomalies in the dorsal indentation of the facial nerve (n=12), local thickening of arachnoid adhesions (n=6), and several other coexisting anatomical abnormalities (n=3) (**Table 1**), totaling 42 cases with exposure or discrimination difficulties under simple microscopic visualization. Endoscopic-assisted vascular exploration and decompression was therefore required.

**Table 1:** Anatomical abnormalities involved in hemifacial spasm patients.

Anatomical abnormalities	n
Abnormal fullness of the cerebellar flocculus	37
Narrow posterior fossa	9
Offending vessels in the outer peripheral portion of the facial nerve with protrusion of abnormal bone	3
Complex vascular anomalies in the dorsal indentation of the facial nerve	12
Local thickening of arachnoid adhesions	6
Several other coexisting anatomical abnormalities	3

### Preoperative preparation

**Figure 1:**
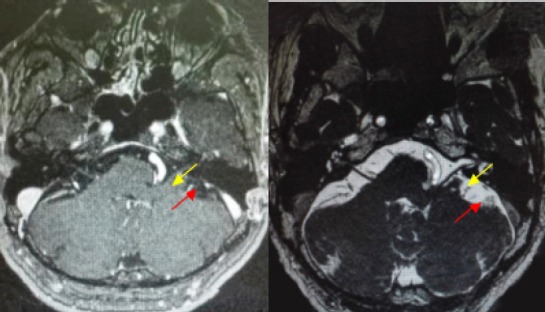
- The patient was 42 years old, female, MR showed that the facial nerve was associated with vertebral artery, but it is not clear. (yellow arrow showed vertebral artery; red arrow showed facial nerve)

All patients underwent enhanced MRI and magnetic resonance angiography (MRA) to understand the relationship between the auditory nerve and responsible blood vessels for development of the operation plan (**Figure 1**). A rigid 30-degree endoscope with 4-mm outside diameter (asap endoscopic products GmbH, Umkirch, Germany) was used in all cases. Endoscopic images were viewed and recorded using a Stryker optical system (Stryker Inc., Kalamazoo, MI, USA) and Sony video recorder and color monitor (Sony Electronics Inc., Tokyo, Japan). A rigid endoscope with 0- and 30-degree viewing lenses (Storz Medical AG, Tägerwilen, Switzerland) and related monitoring systems were used intraoperatively (Aesculap Inc., Center Valley, PA, USA).

### Surgical methods

**Figure 2:**
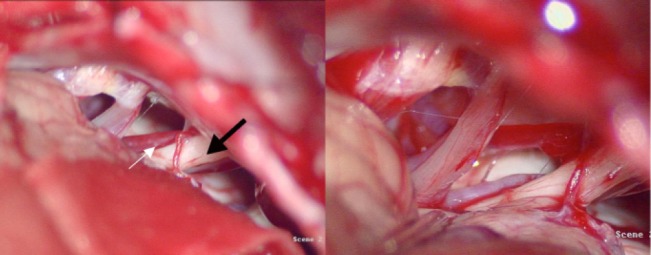
- Microscopic view in hemifacial spasm surgery **a)** A parallel vessel is found at the medial side of the facial nerve and believed to be the offending vessel. However, the scope of contact and degree of compression cannot be identified (black arrow: acoustic nerve; white arrow: medial vessel), **b)** Exposure remains unsatisfactory even after the flocculus is retracted.

**Figure 3:**
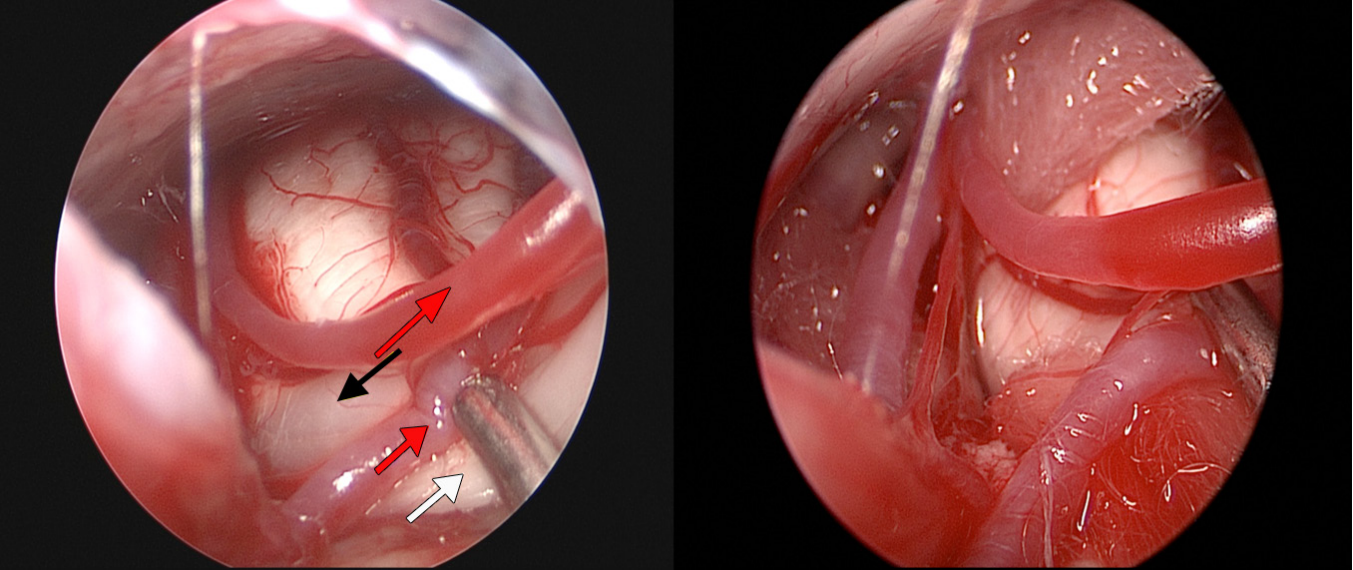
- Endoscopic view in hemifacial spasm surgery **a)** The root exit zone (REZ) of the facial nerve and its relationship with vessels can be visualized under endoscopy (yellow arrow: REZ of the facial nerve; white arrow: acoustic nerve; red arrow: offending vessels. **b)** A Teflon pledget was used on both sides of the facial nerve in a bridge-shaped form in order to separate the offending vessels, thus achieving satisfactory decompression.

A suboccipito-retrosigmoid approach was used, with a decompression-window diameter of 2–3 cm. The margin between the window and the transverse sinus and sigmoid sinus junction was approximately 1 cm. Craniotomy procedures were the same as in ordinary MVD surgery. Under the operative microscope, CSF was slowly released in order to probe and reveal the facial nerve. In the event of a significant blind spot, which is seen under the microscope, in the event of a cerebellar flocculus that is too full, petrous bone-protrusion abnormalities, significant choroid plexus blockage, excessive thickening of arachnoid adhesions, an overly complicated, or even completely dorsally located, anatomy of the responsible vasculature (**Figures 2A, 2B**) endoscopic exploration was performed (**Figure 3A**) with the lens probing along the petrous bone to the cerebellopontine angle to find the facial and auditory nerves (for HFS patients with normal anatomy, it is not necessary to use neuroendoscopy). Under endoscopic vision, multi-angle 360-degree observation was achieved and the relationships between blood vessels and nerves were carefully determined. Facial nerves were fully explored.^8^ This surgical approach brings better results than brain MVD alone, including exploration of the brainstem facial nerve root exit zone (REZ) and exploration of the distal end of the nerve. Microsurgical scissors were used for sharp separation of thickened arachnoid adhesions; after that, the offending vessels were completely pushed away from the facial nerve roots. Appropriate-sized Teflon® polytetrafluoroethylene pledgets (E. I. du Pont de Nemours and Company, Wilmington, DE, USA) were used on both sides of the facial nerve to separate it from the involved vessels in a bridge shape (**Figure 3B**). After surgery, endoscopic examination was performed again to rule out vascular omissions, avascular excessive stretch, kinking, or formation of new compressions. After strict surgical hemostasis and lavage, the dura and skull were closed without placing drains.

## Results

### Local anatomy

During the surgery, abnormal fullness of the cerebellar flocculus was found in 28 cases, and a narrow posterior fossa in 9 cases. With regard to offending vessels, there were 30 cases of anterior inferior cerebellar artery, including 9 cases complicated by other offending vessels, 7 cases of posterior inferior cerebellar artery (including 3 cases complicated by other responsible vessels), and 5 cases of vertebrobasilar artery (including 2 cases involving the lower and/or posterior artery). Of all cases, 12 were entwined with multiple offending vessels and blood vessels in the dorsal indentation. There were 3 cases of offending vessels in the outer peripheral portion of the facial nerve with abnormal petrosal bone protrusion and 6 cases with local thickening of arachnoid adhesions. Blind spots or inability to recognize the offending vessels for one or more of the above reasons were noted in all 42 HFS patients in primary surgery. With the help of adjuvant neuroendoscopy, the offending vessels were clearly identified and complete nerve decompression conducted.

### Surgical effectiveness

With regard to postoperative outcomes, 38 patients were in complete remission, and 4 patients significantly improved, for a total response rate of 100%. In 38 cases, Shorr grade was reduced to grade 0 from grades II or III before surgery, in 3 cases to grade I from grade II, and in one case to grade II from grade IV. Postoperative complications included House–Brackmann grade II facial paralysis (n=2); hearing loss (n=1); and dizziness, nausea, and vomiting (n=4). All symptoms statistically significant improved after one month following neurotrophic, microcirculation, acupuncture, and hyperbaric oxygen therapies. (**Table 2**)

**Table 2:** The complication of endoscopic-assisted MVD and their frequencies (N=42, CN indicates cranial nerve)

Complications	Transient	Permanent
Facial palsy	2 (4.7)	0(0)
Hearing impairment	1 (2.4)	0(0)
Hemorrhage	1 (2.4)	0(0)
Lower CN palsy	1 (2.4)	0(0)
Infection	1 (2.4)	0(0)
CSF leakage	1 (2.4)	0(0)
Others (dizziness, nausea, and vomiting etc)	4 (9.5)	0(0)

### Follow-up

All patients were followed up for 18–30 months. Complete remission without recurrence was seen in 41 cases, 3 cases of discharged patients with grade I recovered to grade 0 levels, and one case of hearing loss was fully restored in 6 months, one case of grade II with no statistically significant increase to the end of follow-up.

## Discussion

Hemifacial spasm is a facial nerve disorder with features of functional hyperactivity that is commonly seen in neurosurgery. Currently a large number of studies have confirmed that pulsatile compression from offending vessels across the brainstem nerve REZ is the most common cause of HFS.^1^ Some authors also believe that in some cases, vascular decompression for facial muscle spasm fails because the offending vessels are not in the facial nerve REZ zone, but are in the inner peripheral portion of the facial nerve channels outside the area of vascular compression.^2^ In such cases, MVD has been the preferred method of treatment. However, for some HFS patients with complex or unusual anatomy (complex HFS patients), MVD alone may have limited effect, and the resulting high recurrence rate has plagued many neurosurgeons. With the rapid development of neuroendoscopic techniques, endoscopic surgery has gradually become an effective complement to MVD for HFS.^3^,^4^

## Limitations of traditional MVD in complex HFS patients

At present, MVD treatment modified by Jannetta^5^ is mostly used for treatment of primary HFS; that is, under the concept of keyhole surgery, the traditional sigmoid sinus approach used for MVD treatment of primary HFS is modified so that the skull open window is set at 2–3 cm, eliminating excessive and unnecessary craniotomy for surgical exposure and thus reducing surgical trauma and postoperative complications. Such surgical methods have a good effect for most HFS patients but can be limited in patients with anatomical variations or complex offending vessels. First, the key to HFS surgery is correct evaluation of the offending vessels to avoid any omission, and all neurovascular contact areas must be screened for adequate nerve decompression. A good surgical field and excellent lighting are essential to a successful operation. At present, the small bone window in MVD surgery will limit the multi-angle viewing range of the microscope. In cases of development of a cerebellar flocculus and poorly exposed facial nerve roots, the operative space is further limited. Second, some HFS patients have complex local neurovascular anatomy, with abnormal local vascular courses or even entwined branches, while offending vessels are located deep in the abnormal vascular plexus. A dead spot in the microscopic view can be created when the cerebellum pompon is so full, and significant abnormal petrosal protrusion into the choroid plexus, enveloped by arachnoid, is present.^1^,^2^ In this case, the cerebellum should be further retracted or excessive separation of the arachnoid will be required to achieve better exposure, which will inevitably result in local excessive neurovascular disruption, cerebellar traction injury, and even avulsion of the petrosal vein, causing serious complications.^6^ Additionally, small offending blood vessels that do not create a pressure trace on the facial nerve or those located on the dorsal side will also impede microscopic evaluation.

## Advantages of neuroendoscopic surgery for complex HFS

Surgical microscope illumination is prone to having a dead corner in the surgical field and does not reveal structures outside the microscope field well.^7^ The neuroendoscope provides excellent lighting and a panoramic viewing angle, as the lens can be inserted into the cranial cavity for full-angle observation of the nerves, brainstem, and blood vessels. This allows identification of the presence or absence of contact with the nerve surface and indentation or presence of blood vessels. At the same time, the entire length of the facial nerve can be viewed as well as the dorsal side,^8^ making it difficult to overlook offending vessels, thus reducing postoperative recurrence. Additionally, endoscopic surgery requires an opening just slightly wider than the width of the lens barrel. The smaller space required prevents stretch irritation to the brain stem, cerebellum, and peripheral nerves and blood vessels, thus greatly reducing surgical complications. Finally, endoscopic surgery allows pre-closure confirmation of appropriate location of the Teflon pads, adequate decompression, and secure placement of pads, providing immediate feedback to the surgeon and significantly improving surgical outcomes.^9^

## Indications for endoscopy

Surgical exposure in the present cohort of 42 patients was challenging because of various anatomic abnormalities that made it difficult to view or recognize the offending vessels. A 0-degree endoscope with no refraction is no different from a microscope (often applied in the Otolaryngology Department, less applied in Neurosurgery), whereas a 70-degree endoscope has such large refraction that it is likely to accidentally injure deep structures (seldom used in the Department of Neurosurgery). In this case, 30-degree-endoscope assistance not only clarifies the offending vessels, but also avoids failure of surgery or iatrogenic injury, thus greatly improving the success rate of surgery in complex HFS patients.

## Operation key points

1. Detailed preoperative planning, including brain MRA or CT angiography, is needed to show the presence of offending vascular compression and rule out location in the cerebellopontine angle and other secondary factors. 2. The head should be correctly placed to be more conducive to revealing the facial nerve REZ region. 3. The anterior border of the bone window should reach the sigmoid sinus, and the lower edge should reach the skull base level. 4. The optical axis must always be kept in line with the microscope field of view and CSF slowly released after opening the dura. When exposure is difficult because of petrosal bone protrusion and fullness of the cerebellar flocculus, the cerebellum must not be forcibly retracted. In such cases, the endoscope can be used to assist the operation in the intraoperative zone structure. 5. Before any sharp cut of the arachnoid, endoscopic observation of the local vasculature is necessary to avoid subsequent relocation of offending vessels that may affect evaluation. 6. Complex HFS patients tend to have a small surgical space and complex internal structures, in particular, when the offending vessel is located in a medial position or hidden, making it difficult to distinguish offending vessels by microscope. The combination of microscopic and endoscopic techniques is important for careful multi angle observation of the facial nerve. 7. Currently, in order to achieve full decompression, we often lift the offending vessels on both sides of the facial nerve root like the shape of a bridge. The use of Teflon pledgets alone is rare nowadays. After surgery, endoscopic confirmation of full and complete padding decompression should be performed.

At the early stage, the most serious complications following MVD in our hospital were cerebellar infarction, edema, tinnitus, hearing loss, and even death, which are associated with over-stretch of the cerebellum.^10^ In recent years, with the assistance of neuroendoscopic techniques, these complications have been significantly reduced and no case of secondary brain contusion, cerebral infarction, or hearing loss has ever occurred.^11^,^12^ However, the rates of facial paralysis, CSF leakage, and delayed healing are similar to those in simple MVD.

## Limitations of endoscopy

All HFS patients in this study underwent neuroendoscopic-assisted separation and decompression, but we believe that while neuroendoscopy is an effective tool,^13^-^15^ not all HFS patients require endoscopy. The three-dimensional view and excellent depth of field of the microscope have their inherent advantages. It is still preferred for those with a large posterior fossa volume, no significant cerebellum pompons or petrosal shelter, fully released CSF, and clearly identified offending vessels. Although neuroendoscopy has its obvious advantages, there are disadvantages that cannot be ignored. First, the endoscope takes up most of the bone window, making it impossible to see bilateral and posterior structures after entry into the head, which may risk damage to the surrounding structures when changing positions or angles. Second, endoscopy can provide only a two-dimensional image, which lacks a sense of depth. Most neurosurgeons are not familiar with endoscopic imaging and manipulation techniques, as opposed to entirely endoscopic MVD. Many physicians prefer endoscopic-assisted MVD. Third, surgeons need to support the mirror while performing the surgery, which can lead to operational difficulties. At the same time, there is still no specific surgical instrument for endoscopic treatment of subarachnoid lesions. Although a 30-degree endoscope can be used to observe some lesions, it does not allow operation. Additionally, bleeding can easily contaminate the endoscope lens, affecting image quality and increasing operational difficulty, thus affecting the coherence of the procedure. In addition, the heat generated by the light source and the sharp edges of the endoscope are also likely to injure nearby structures.

In conclusion, endoscopy is a useful complement to traditional nerve MVD surgery.^16^ The advantage of endoscopy lies in its excellent lighting and panoramic perspective, while microscopy provides three-dimensional stereoscopic advantages and excellent depth of field. Combining the 2 with their respective advantages, may facilitate and improve surgery in complicated HFS patients.
